# Detection of Bisphenol A and Four Analogues in Atmospheric Emissions in Petrochemical Complexes Producing Polypropylene in South America

**DOI:** 10.3390/molecules27154832

**Published:** 2022-07-28

**Authors:** Joaquín Hernández Fernández, Yoleima Guerra, Heidi Cano

**Affiliations:** 1 Chemistry Program, Department of Natural and Exact Sciences, San Pablo Campus, University of Cartagena, Cartagena 130015, Colombia; 2 Centro de Investigación en Ciencias e Ingeniería, CECOPAT&A, Cartagena 131001, Colombia; yoleima.guerra@cecopat.com; 3 Department of Civil and Environment Engineering, Universidad de la Costa, Barranquilla 080002, Colombia; hcano3@cuc.edu.co

**Keywords:** bisphenol A, bisphenol analogues, emissions, polypropylene

## Abstract

Because of its toxicity and impacts on the environment and human health, bisphenol A (BPA) has been controlled in numerous industrialized nations, increasing demand for bisphenol analogues (BP) for its replacement. However, the consequences of these chemicals on the environment and the health of persons exposed to their emissions are still being researched. The emissions from polypropylene manufacturing facilities in Colombia and Brazil were evaluated in this study, and the presence of bisphenol A and four BPs was detected among the gaseous compounds released, with total concentrations of BPs (∑BP) between 92 and 1565 ng g^−1^. As the melt flow index (MFI) of the polymer rises, so does the quantity of volatiles in its matrix that are eliminated during deodorization, indicating that the MFI and the amount of bisphenol released have a directly proportional connection.

## 1. Introduction

Bisphenol A (2,2-bis-(4-hydroxyphenyl)-propane; BPA), its derivatives, and analogues are chemical products that are part of the compounds with two hydroxyphenyl functional groups in their chemical structure [1]. With a production of more than 5 million tons per year, BPA is one of the most produced products among bisphenols (BPs) [2] and is widely used as a raw material in various plastics (powder paints, polycarbonate plastics, epoxy resins, and paper coatings). However, BPA can be considered as one of the most important industrial additives in the production of resins; it is a highly polluting substance mainly generated by effluents from the plastic industry, leachate from plastic waste deposited in landfills, electronic waste, industrial painting, and BPA production [3]. This chemical, together with toxic dyes and nonylphenol, is filtered into the environment due to the natural degradation and decomposition of synthetic plastics, reaching concentrations of BPA of up to 100 mg L^−1^ in water at lethal concentration 50 (LC50) or effective concentration 50 (EC50) of 10 mg L^−1^ in water effluents from plastic production activities [4,5].

The effects of BPA on human health and the environment have been extensively studied, leading to the determination that this substance is responsible for disrupting the normal functions of hormones [6]. These compounds are known as endocrine-disrupting chemicals (EDC), since they are responsible for altering the functioning of estrogenic and androgenic hormones and are commonly found among the contaminants identified in soils and in bodies of water [7]. The negative effects of bisphenol A on the development of the human neuronal, cardiovascular, immune, and metabolic systems have also been reported [8,9], which is why the use of this substance has been limited or prohibited in many countries, and bisphenol analogues have been implemented as a solution to this problem [1,10]. However, these compounds have been considered toxic; several studies have evaluated their effects on human health, and it is considered a possible genotoxin. It has even been evidenced in human blood, urine, and breast milk, evidencing the long exposure they have inadvertently had to this compound [11,12,13]. Therefore, the identification and quantification of new sources of generation and new derivatives of BPA not reported in the literature are important in order to have a greater inventory of their sources of origin and propose greater regulations for their application. Several studies have investigated the occurrence and profiles of BPs in sediments, indoor dust, paper products (e.g., currency), and human urine [14,15]. Reports of ambient levels of BPs in gaseous emissions from PP-producing plants are scarce, focusing on countries such as the United States, China, Japan, and Korea [1,16].

Colombia and Brazil are countries that have polypropylene production plants which have been previously studied for the detection, identification, and quantification of the concentration of contaminants that are of interest and can affect the health of the surrounding inhabitants [17,18,19,20,21,22,23,24]. This allows us to observe that the development of analytical methods to identify and quantify other types of contaminants, such as bisphenols, for example, is becoming increasingly relevant in order to publicize the levels of these contaminants, which can subsequently be regulated and controlled to guarantee greater sustainability of the regions. It is imperative to assess the contribution of BP emission pathways to the environment in order to delineate the environmental fates, risks, and management of these chemicals.

In this study, gaseous emissions from polypropylene production plants in Colombia and Brazil are evaluated to identify the presence of BP. The samples were taken during the production of polypropylene with different melt flow indexes to determine if this influences the amount of BPs emissions during the removal of volatile compounds in the deodorization column, using analysis techniques such as solid phase extraction (SPE) and high-performance liquid chromatography (HPLC) for the detection of bisphenols.

## 2. Materials and Methods

### 2.1. Sampling Sites

**Figure 1:**
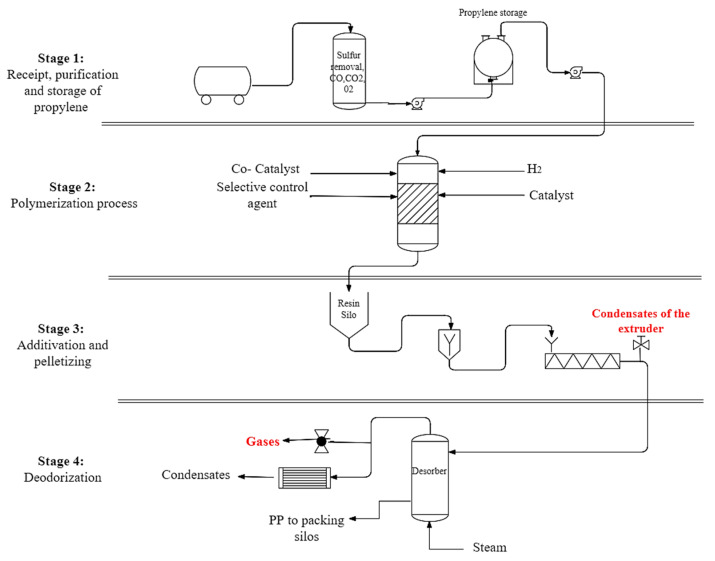
Polypropylene production stages and sampling points.

The selected polypropylene plants are situated in the industrial zones of Colombia and Brazil. The four key steps of the manufacturing process of PP are: 1. reception, purification, and storage of raw materials; 2. polymerization; 3. addition, extrusion, and pelletizing; and 4. desorber, as shown in Figure 1. The study focuses on the fundamental processes of deodorization. The gaseous samples are collected at the top of the desorber. The desorber works with water vapor ranging from 600 to 1200 kg h^−1^ and steam ranging from 120 to 140 °C.

### 2.2. Sample Collection 

The study was conducted during the manufacturing of three classes of PP with MFIs of 5, 50, and 80. Polypropylene samples with this MFI were used because it was shown in previous studies that the value of this characteristic of PP influences the amount of volatile compounds present in the polymeric matrix, following the methodology explained by J. Hernández (2020) [17].

The desorber produced effluent from the desorption of six grades of PP. Because of the construction of the column, each grade of PP took 4 h to desorb in the desorber. To have a better understanding of the stability of the process, samples were obtained in triplicate every hour. This allowed the variability of the desorber to be evaluated, as well as the influence of time spent in different phases of the process and the migration of the phenols to the condensates.

One meter away from the highest point of the column is where the sample point for the desorber is situated. This point features a nipple and a steel tube in the shape of a flute with a longitude equal to the diameter of the column. This flute features 20 evenly spaced orifices, allowing sampling of more than 95% of the diameter of the column. The end of the flute has two outlets, one of which is attached to a vacuum pump that operates at a constant pressure and the other to a metal cylinder that is coated in sulfurite. With the help of this system, it is possible to collect an isokinetic sample such that each of the 20 orifices receives the same quantity of sample due to equal pressure. A representative isokinetic sample is ensured by the 20 orifices, which allow more than 95% of the laminar flow of gases from the column to enter. The gases are mingled within the flute of each orifice before being later collected into the sulfurite-lined interior of the steel cylinder. For this investigation, a total of 72 gaseous samples—each obtained in triplicate—were used. The desorber allowed each grade of PP to desorb for 4 h.

### 2.3. Instrumental Analysis

**Figure 2:**
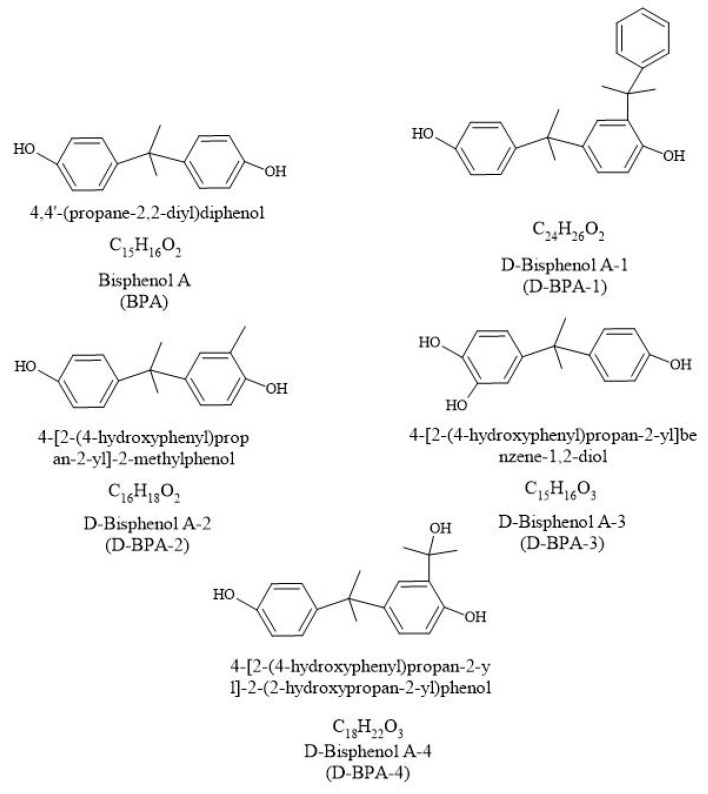
Structures and identifications of the BPs of interest.

BP structures of interest are shown in Figure 2. For the development of the analysis, the procedure of Sunggyu L. et al. (2015) [25] was followed. An Applied Biosystems API 2000 electrospray triple quadrupole mass spectrometer (ESI-MS/MS; Applied Biosystems, Foster City, CA, USA) and an Agilent 1100 Series HPLC (Agilent Technologies Inc., Santa Clara, CA, USA) outfitted with a binary pump and an autosampler were used to measure the concentrations of BPs in sample extracts. For LC separation, a Javelin guard column (Betasil C18, 20 × 2.1 mm) was attached to an analytical column (Betasil C18, 100 × 2.1 mm; Thermo Electron Corporation, Waltham, MA, USA). The injection has a 10 L volume. With a gradient as follows, the mobile phase consisted of methanol and water flowing at a rate of 0.3 mL min^−1^: 15 percent methanol for the first two minutes; 15–50 percent methanol for the next five minutes; 50 percent methanol for the next eight minutes; 90–99 percent methanol for the next twenty minutes; and 15 percent methanol for the final thirty minutes. It was performed using the negative ion multiple reaction monitoring (MRM) mode. By injecting several substances into the mass spectrometer using a flow injection technique, the MS/MS settings were tuned. Both the impact gas and the curtain gas were nitrogen. The quantification was performed using an external calibration technique, and it was adjusted for C12-BPA recoveries [25].

### 2.4. Treatment of Samples Based on SPE

#### 2.4.1. Pretreatment

The work sample was blended and cooled at 25 °C before being filtered through a 0.22 m PTFE Teflon filter to aid in further sample preparation and limit microbial activity.

#### 2.4.2. Preconcentration and Cleaning

Conditioning of Strata X-33 cartridges (6 mL, 500 mg) was conducted at this step using 5 mL of MeOH followed by 5 mL of distilled water. After that, 15 mL of the material was uploaded at a rate of 1 mL per minute. After percolating the entire sample, the cartridges were rinsed with 3 mL of MeOH:H_2_O 80:20. The chemicals retained in the solid phase were eluted with 10 mL of ACN. The eluate was evaporated until dry using a nitrogen stream at 5 psi. The finished extract was reconstituted with ACN to a final volume of 1 mL, yielding a 10:1 pre-concentration [17].

### 2.5. Quality Assurance and Quality Control (QA/QC)

Spiked blanks and matrix-spiked samples were frequently tested to confirm the analytical approach utilized in our investigation. BP recoveries in spiked blanks (*n* = 4) varied from 75.4 to 1.44 percent (mean SD) for D-BPA-1, and 256 to 1.32 percent for D-BPA-2 (30 ng for each compound and 20 ng for C_12_-BPA). BP recoveries in matrix-spiked samples (*n* = 4) varied from 45.3 to 1.7 percent for D-BPA-3 and 325 to 5.14 percent for D-BPA-4 (30 ng for each individual component and 20 ng for C_12_-BPA). The lowest admissible calibration standard and a notional sample weight of 0.1 g were used to compute the limit of quantitation (LOQs). The computed LOQs for BPA, D-BPA-1, and D-BPA-2 were 0.40 ng g^−1^; 0.9 ng g^−1^ for D-BPA-3; and 1.5 ng g^−1^ for D-BPA-4. After every 20 samples, a midway calibration standard was injected to assess for drift in instrumental sensitivity. A pure solvent (methanol) was injected at regular intervals to check for BP carryover between the samples examined. The daily injection of 10 calibration standards at concentrations ranging from 0.01 to 250 ng mL^−1^ confirmed the instrumental calibration, and the linearity of the calibration curve (r) was more than 0.99 for each of the target compounds. BP concentrations are reported as a dry weight (dw). 

To perform statistical analysis, concentrations below the LOQ were assigned a value of one-half of the corresponding LOQ rather than a zero value for the computation of the mean and median. To investigate variations in BP concentrations across three distinct PP types, a one-way ANOVA with Tukey’s test was used. To analyze differences in BP concentrations throughout the manufacturing of three classes of PP with fluidity indices (MFI) of 5, 50, and 80, a one-way ANOVA with Tukey’s test was used [25].

## 3. Results

### Occurrence and Concentrations of BPs in Atmospheric Emissions

BPs concentrations in atmospheric emissions collected from PP production plants in Colombia and Brazil are summarized in Table 1. The sampling points were established with alphanumeric codes that contained the letter of each country and the MFI of interest. For Colombia, the sampling points for BP analogue emissions were EC-MFI5, EC-MFI50, and EC-MFI80, thus guaranteeing the taking of gaseous samples when this plant was producing PP with an MFI of 5, 50, and 80, respectively. For the sampling of emissions at the plant in Brazil, points EB-MFI5, EB-MFI50, and EB-MFI80 were selected. The concentrations of total BPs (BPs; the sum of 5 bisphenol analogues) ranged from 92 to 1565 (average: 628) ng g^−1^ dw. BPA and all its analogues presented detection rates (RD) of 100% in all the samples studied, and this was independent of the type of MFI of PP that was synthesized. The average concentrations of the BPs detected in simple emission gas were in the order of BPA (338.56 ng g^−1^ dw), D-BPA-1 (131.32 ng g^−1^ dw), D-BPA-2 (51.97 ng g^−1^ dw), D-BPA-3 (62.07 ng g^−1^ dw), and D-BPA-4 (42.67 ng g^−1^ dw), indicating that BPA is a by-product of the degradation of PP in this type of industrial process. Between the two sampling points in Colombia and Brazil and under the three different sampling conditions at each plant, the highest concentrations of BPA were found in the industrial emissions from the Brazil plant during the production of PP with an MFI of 80 (mean: 980.59 ng g^−1^ dw), followed by the emissions of this same plant with a production of MFI of 50 (708.11 ng g^−1^ dw) and the emissions of the Colombia plant when it produced PP with an MFI of 80 (670.83 ng g^−1^ dw). Our results suggest that environmental contamination by BPs in South America has a very significant contribution from industrial activities rather than domestic activities and, therefore, corrective measures must be taken in a very short time. The concentrations of BPA, D-BPA-1, and D-BPA-3 in all gas emissions samples from PP production plants in South America ranged from 45 to 947 (average: 338.556) ng g^−1^ dw, from 15 to 542 (average: 131.32) ng g^−1^ dw, and from 16 to 124 (average: 62.07) ng g^−1^ dw, respectively.

In both the Colombian and Brazilian plants, the concentrations of BPA and its analogues varied significantly in the PP production samples, with MFIs of 5, 50, and 80. The fluidity and crystallinity of the material increases as the MFI value increases. The PP of MFI of 80 has shorter chains in its structures, and this makes the content of volatiles in its polymeric matrix higher. Therefore, it would be expected that during the PP deodorization process, additive residues or PP degradation by-products can be more easily removed from the polymer matrix. The results allow us to observe that the means of the total emissions of BP in Colombia were 389.07, 487.72, and 670.83 ng g^−1^ dw, respectively, being directly proportional to the MFI values. In the Brazilian plant, the same relationship of BP with MFI was also observed, except that the concentrations of BPs were higher at 523.28, 708.11, and 980.59 ng g^−1^ dw, respectively. 

**Figure 3:**
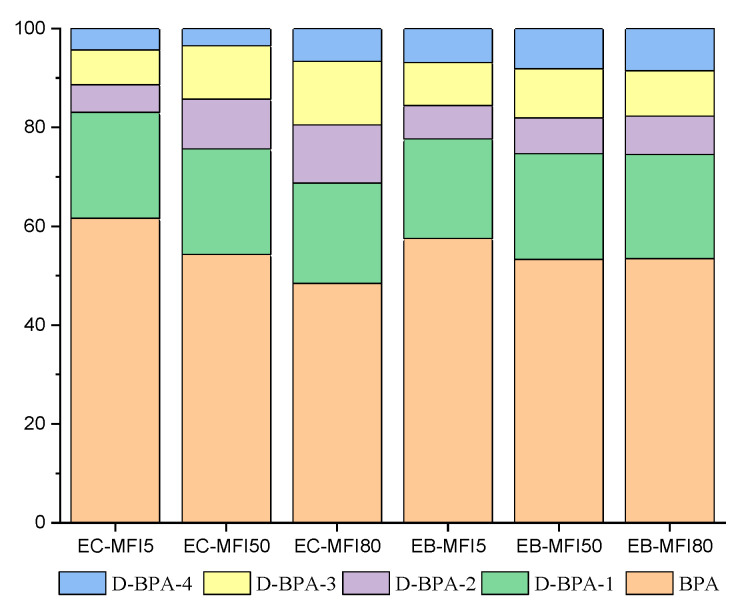
Comparisons of the % of BPA, D-BPA-1, D-BPA-2, D-BPA-3, and D-BPA-4 in Colombia and Brazil.

Concentrations of BPA, D-BPA-1, D-BPA-2, D-BPA3, and D-BPA-4 were 9.5, 19.95, 55.62, 48.19, and 0% higher, respectively, in emissions from the Colombia plant during production of a PP MFI50 compared to the production of a PP MFI5, as seen in Figure 3. Regarding the production of MFI80, the increases were 26.19, 38.91, 72.30, 68.45, and 61.87% higher than the emissions in MFI5. These significant differences could be appreciated when comparing only the variation in the fluidity of the PP. However, when comparing the plant in Colombia with the one in Brazil, we also noticed important differences, and this can be seen for each BPA analogue. In this way, the results in the plant in Brazil were always 20% higher than in the plant in Colombia. For MFI5, the values of BPA, D-BPA-1, D-BPA-2, D-BPA3, and D-BPA-4 were 20.37, 21.07, 37.85, 40.23, and 52.85% higher, respectively, in the Brazilian plant. During the production of MFI50, differences greater than 25% were observed for BPA, D-BPA-1, D-BPA3, and D-BPA-4, while for D-BPA2, the difference was only 3.89%. During the production of MFI80, emissions in Brazil exceeded 34% for BPA, D-BPA-1, and D-BPA-4, while the difference for D-BPA2 and D-BPA3 was only 3.65 and 3.78%, respectively.

The values obtained in bisphenol emissions have not been reported before for petrochemical plants. However, when compared with the data obtained in other studies of bisphenol emissions in homes, offices, and urban and residential areas, it is evident that the results obtained are superior to those obtained in Greece, Spain, and Romania [26,27,28]. In a study carried out by Wang et al. (2015) [26], dust samples were taken inside houses in different countries, including Colombia, which obtained values similar to those reported (500 ng g^−1^).

The values obtained in this study are worrying. These emissions are made every day of production and are dispersed in the environment, affecting the surrounding population without their knowledge. There are no regulations in these countries on the limit of exposure and emission of BPs allowed to control these compounds. These studies are important because there are no controls on these components. In addition, these companies do not record the BPs concentrations found in this study, which indicates that they may be a product of the decomposition of additives during the production of polypropylene.

## 4. Conclusions

This is the first industrial-scale investigation on the detection of bisphenols in the polypropylene process. This enables the evaluation of the phases and equipment where bisphenol emissions occur. Furthermore, the detection of compounds similar to bisphenol A makes it possible to evaluate the effects of these bisphenol A substitutes on humans in industrial areas, since the concentrations found and constant exposure to it can cause harmful effects in workers, and protocols to ensure their health are recommended.

## Figures and Tables

**Table 1:** Concentrations of bisphenol analogues (ng g^−1^ dry weight) in atmospheric emissions from PP production plants in Colombia and Brazil.

Country	Sampling Point	Parameters	BPA	D-BPA-1	D-BPA-2	D-BPA-3	D-BPA-4	∑BPs
Colombia	EC-MFI5	Production MFI 5 (*n* = 16)						
		Mean	239.71	83.29	21.81	27.29	16.97	389.07
		Median	214	64	16.7	25.9	13.2	393.7
		Range	45–546	15–341	0.57–81.6	15.6–43.5	4.26–51.6	92.45–976.75
		DR	100	100	100	100	100	100
	EC-MFI50	Production MFI 50 (*n* = 16)						
		Mean	264.88	104.06	49.14	52.68	16.97	487.72
		Median	246	88	46.6	49.4	13.2	510.9
		Range	73–576	26–421	1.85–134.5	29.3–75.3	4.26–51.6	160.16–1129.15
		DR	100	100	100	100	100	100
	EC-MFI80	Production MFI 80 (*n* = 16)						
		Mean	324.76	136.35	78.72	86.51	44.49	670.83
		Median	345	99	76.9	91.6	34.6	698.6
		Range	95–624	42–542	6.5–172.5	46.7–121.1	19.4–99.7	337.6–1383.4
		DR	100	100	100	100	100	100
Brazil	EB-MFI5	Production MFI 5 (*n* = 16)						
		Mean	301.01	105.53	35.09	45.66	35.99	523.28
		Median	316	87	34.6	42.1	33.9	530
		Range	79–615	34–369	2.43–99.3	31.2–74.3	9.75–81.3	186.75–1123.2
		DR	100	100	100	100	100	100
	EB-MFI50	Production MFI 50 (*n* = 16)						
		Mean	377.12	151.71	51.13	70.41	57.75	708.11
		Median	419	141	46.5	66.5	55.6	699.8
		Range	99–721	49–455	4.65–112.1	47.6–99.2	25.4–98.4	308.05–1339.7
		DR	100	100	100	100	100	100
	EB-MFI80	Production MFI 80 (*n* = 16)						
		Mean	523.88	207.00	75.95	89.90	83.86	980.59
		Median	536	196	75.3	88.5	77.2	1002.6
		Range	184–947	75–523	10.8–201.5	66.2–124.3	46.5–142.5	430.5–1565.1
		DR	100	100	100	100	100	100
**Total (*n* = 40)**								
Mean			338.56	131.32	51.97	62.07	42.67	626.60
Median			294	99	44.35	64.8	35.5	542.95
Range			45–947	15–542	0.6–201	16–124	4–142	92–1565
DR			100	100	100	100	100	100

## Data Availability

Not applicable.
